# Rapid evidence synthesis to enable innovation and adoption in health and social care

**DOI:** 10.1186/s13643-022-02106-z

**Published:** 2022-11-23

**Authors:** Gill Norman, Paul Wilson, Jo Dumville, Peter Bower, Nicky Cullum

**Affiliations:** 1 Division of Nursing, Midwifery & Social Work, School of Health Sciences, Faculty of Biology, Medicine & Health, University of Manchester, Oxford Road, Manchester, M13 9PL UK; 2 NIHR ARC GM, Centre for Primary Care and Health Services Research, School of Health Sciences, Faculty of Biology, Medicine & Health, University of Manchester, Manchester, UK; 3 Manchester Academic Health Science Centre, Research and Innovation Division, Manchester University Foundation NHS Trust, Manchester, UK

## Abstract

**Background:**

The rapid identification and adoption of effective innovations in healthcare is a known challenge. The strongest evidence base for innovations can be provided by evidence synthesis, but this is frequently a lengthy process and even rapid versions of this can be time-consuming and complex. In the UK, the Accelerated Access Review and Academic Health Science Network (AHSN) have provided the impetus to develop a consistently rapid process to support the identification and adoption of high-value innovations in the English NHS.

**Methods:**

The Greater Manchester Applied Research Collaboration (ARC-GM) developed a framework for a rapid evidence synthesis (RES) approach, which is highly integrated within the innovation process of the Greater Manchester AHSN and the associated healthcare and research ecosystem. The RES uses evidence synthesis approaches and draws on the GRADE Evidence to Decision framework to provide rapid assessments of the existing evidence and its relevance to specific decision problems. We implemented this in a real-time context of decision-making around adoption of innovative health technologies.

**Results:**

Key stakeholders in the Greater Manchester decision-making process for healthcare innovations have found that our approach is both timely and flexible; it is valued for its combination of rigour and speed.

Our RES approach rapidly and systematically identifies, appraises and contextualises relevant evidence, which can then be transparently incorporated into decisions about the wider adoption of innovations. The RES also identifies limitations in existing evidence for innovations and this can inform subsequent evaluations. There is substantial interest from other ARCs and AHSNs in implementing a similar process. We are currently exploring methods to make completed RES publicly available. We are also exploring methods to evaluate the impact of using RES as more implementation decisions are made.

**Conclusions:**

The RES framework we have implemented combines transparency and consistency with flexibility and rapidity. It therefore maximises utility in a real-time decision-making context for healthcare innovations.

**Supplementary Information:**

The online version contains supplementary material available at 10.1186/s13643-022-02106-z.

## Introduction

### Rapid evidence synthesis

Whilst evidence synthesis can represent the strongest evidence base for innovations, conventional systematic reviews may often take up to 2 years to produce [1, 2], whilst even rapid reviews have a timeframe which may range up to a year [3], with the extent to which methods differ from those of systematic reviews varying widely [4]. Evidence summaries or evidence briefings are a form of rapid evidence synthesis which are usually produced on a shorter timescale driven by decision-makers’ needs [5] and have been found useful in informing decision-making, including by sub-national healthcare administrations [6]. Rapid evidence synthesis (RES) has been used to inform the commissioning of research [7] and services [8] and to inform policy-making [9, 10]. However, a standardised process of rapid evidence synthesis has not yet been presented to inform decision-making around accelerating access to beneficial innovation adoption, where timescales are often short. We present the process we have developed to enable integrated evidence synthesis within such a decision-making context.

### Background and context

Challenges in getting proven innovations rapidly adopted into systems, policies or practice have long been recognised. In the UK, the Accelerated Access Review provided fresh policy impetus to efforts to develop a faster pathway to identify and adopt high-value innovations in the National Health Service (NHS) in England [11]. The Accelerated Access Review set out recommendations to improve efficiency and outcomes for NHS patients by increasing the speed of access to beneficial innovative healthcare methods and technologies, including digital products. A key part of increasing access has been the development of infrastructure including the Academic Health Science Networks (AHSNs) and the NHS Accelerated Access Collaborative (AAC). AHSNs are the agencies charged with supporting the introduction and diffusion of innovative products across the NHS.

In Greater Manchester, Health Innovation Manchester (HInM) is the AHSN with the remit to implement beneficial health innovations across the region [12, 13]. Our definition of innovation includes any technology, device, procedure, set of behaviours, routine or way of working that is new to the Greater Manchester context [14].

Decisions about innovation adoption may need to be made rapidly but this does not negate the need for evidence-informed decision-making, although for some novel technologies the available evidence may be limited. It remains important that there should be transparency about the evidence used, its reliability and relevance to the decision problem and what, if any, further evaluation may be warranted.

### Rapid evidence synthesis in Greater Manchester

To ensure that decisions in Greater Manchester on innovation adoption and roll-out are informed by evidence, we developed a framework for the production of RES for innovations being considered for implementation. This framework has been made publicly available and registered [15] and is provided here, together with a representative example (see Supplementary material). The framework builds on earlier work and experience in developing frameworks for evidence briefings including those to directly inform decision-making by healthcare organisations [16–18]. It also has some similarities to other evidence briefing approaches which were identified in previous work [19]. However, the approach we have developed is unique in several important respects, which are described here, and represents a RES development in combining the key considerations of speed, transparency, dual emphasis on robustness and relevance of evidence [20, 21] and usability for stakeholders in the context of decisions on innovation adoption.

#### Speed and flexibility

The RES we produce are designed to be requested, undertaken, and delivered in a time period of 2 weeks. We use a systematic but streamlined process to enable replicable, transparent yet consistently very rapid delivery compared with other rapid evidence synthesis processes [6–9]. Our RES approach is also explicitly designed to take account of the fact that the evidence for innovations may be sparse or of limited relevance and incorporates protocols for dealing with innovations which are complex interventions [22, 23]. Flexible question sets have provisions both for category-level appraisal of the evidence or for component analysis of innovations [24]. This means that even where there is very limited evidence for an innovation per se, the findings of a RES using our approach can still inform implementation decisions. We explore examples of this in the section below on “Flexibility in structuring the rapid evidence synthesis*”*.

#### Integration

The production of RES is embedded within, and integral to, the Greater Manchester innovation adoption decision-making process, rather than representing an input from a separate organisation. This integration means that key stakeholders and reviewers can work closely together to co-produce the RES. HInM uses a high-level, broad framework which involves identifying and prioritising innovations which are assessed and selected via an ongoing “pipeline” process [25], whereby support is provided for the roll-out of innovations across the region, where those innovations have been identified as a priority by stakeholders [26]. Innovations may come via academia or industry but may also be flagged as being a high priority from a local, regional or national perspective by local or national decision-making bodies. We refer here to the organisation which has identified or developed the intervention as “the sponsor”. The process draws on ARC and AHSN expertise in implementation science, healthcare decision-making and lived experience, as well as evidence synthesis and evaluation. The innovations for which we conduct RES are those which are submitted to this process and meet initial criteria as potentially relevant innovations.

The RES team attends relevant meetings where innovations are discussed, and the need for RES is explored on a case-by-case basis. Where a RES is requested, the review plan is developed iteratively between reviewers and decision-makers. This enables resolution of queries at each stage, maximises the relevance of the RES and supports integration of the evidence appraisal into decision-making.

RES is recognised as one of the necessary components of the early stage of the decision-making process around innovation adoption, alongside public patient involvement and engagement (PPIE) input, business case assessment and consultation with local health and social care stakeholders. The assessment of evidence, its relevance and certainty, is integrated into the considerations before decisions are made as to whether to proceed with the development of the implementation plans. This consideration of evidence may include wider evidence about the impact of interventions for similar decision problems. The RES does not produce recommendations, and decisions are not determined solely by the findings of the RES, but the RES ensures that decisions are evidence informed. In a small number of instances, the RES proves critical to the decision, usually where a decision is made not to proceed. For example, one RES identified that the innovation was addressed by a national guideline that recommended that a particular intervention should not be offered. Usually the contribution is more nuanced, however.

#### Transparency and consistency

The principles of the GRADE evidence to decision framework are central to our approach to RES, which makes the RES, and the decision process to which it contributes, more transparent, consistent and reproducible [20, 21]. GRADE provides a clear set of considerations for the formation of judgements about the strength of the evidence base for each question addressed and is central to the consideration of the certainty and relevance of the evidence, which are inter-related. The available GRADE frameworks have undergone substantive developments since our previous work on evidence briefings [16].

#### Relevance

The RES uses GRADE approaches to consider the applicability of the evidence to the Greater Manchester context as well as its reliability. Context, both broadly and narrowly considered, can be key to the impact of introducing an intervention [27]. In the cases of complex or service-level interventions, in particular, it can be difficult to determine the boundary between the intervention and the context [28]. For example, a test for poor prognosis in heart failure [29] has potential implications for an entire treatment pathway [30], emphasising the importance of adopting a test-and-treat approach [31]. We are also mindful of the fact that many innovations may look superficially simple but are being considered for introduction into complex systems such as primary care [32].

Although in the first instance our RES consider relevance in terms of a UK-wide NHS context, we also consider the local Greater Manchester context. We do not generally undertake RES where an innovation is already a nationally mandated priority, so local context is potentially important to all the innovations assessed. Local context includes the existing service models, relevant infrastructure, and area and population characteristics including urbanicity, relative deprivation, etc. GRADE helps to ensure that relevance can be given equal prominence to certainty in evidence summaries. An example of identified limited relevance at the national level is where an RES of an innovation in asthma care identified only evidence from US trials. This is not directly relevant to asthma control in people with asthma in the UK, who at the population level have higher baseline control and use of preventative medication [33]. At the local level, an RES for an innovation improving connections between healthcare staff included evidence from a pilot project where one site was very rural [34, 35]. The evidence from that site was considered likely to be only indirectly relevant to Greater Manchester.

### Process and stages of RES

We present a full example of an RES in the Supplementary material.

The key elements of the completed RES are shown in Table 1.

**Table 1:** Structure of RES

• A headline summary of the certainty and relevance of available evidence • A bulleted summary for each question addressed • A description of the innovation • A set of key questions • A summary of the search process including the search date and key terms • Detailed answers to each question addressed in terms of available evidence and its certainty and relevance • Bibliography of sources used to answer the questions	

#### Timeframe and personnel

The RES is designed to produce a “good-enough” (rather than perfect) summary of the evidence as a contribution to decision-making in a short timeframe. “Good-enough” is considered to be an up-to-date evidence summary which is based on the most relevant and methodologically rigorous evidence available, with uncertainties clearly articulated and acknowledged. The methods described are implemented using a median of up to 2 days of time for an experienced researcher with a background in evidence synthesis, spread over an approximately 2-week period. More complex innovations may entail more resources for RES production and may involve more input from an information specialist. More details are provided in the framework (see Supplementary information) [15].

#### Describing the innovation

The first stage is to briefly describe the innovation in terms of its nature and purpose (Table 2). This establishes the type of innovation (e.g. intervention/test/service delivery mechanism), the population or system that is targeted and the outcomes which should be considered. A comparator, which is usually the current standard of care, is typically also identified through this process. This stage involves assessment and clarification of the information supplied by the sponsor through engagement with HInM and/or other relevant stakeholders. This includes determination of the key characteristics of the innovation which will inform the decision problem.

**Table 2:** Example: innovation description

**Phagenyx** is a device which is designed to reduce neurogenic dysphagia (dysfunction of eating) [1]. This is dysphagia arising from the disruption of any of the neurological systems or processes involved in the execution of a coordinated safe swallow and occurs in people following stroke and in other conditions such as multiple sclerosis which impact muscle control. Dysphagia also occurs in people who have undergone sustained intubation for any reason. Phagenyx is classed as a pharyngeal electrical stimulation intervention and comprises a base station and a treatment catheter. It is applied over a period of days. NICE guidance on the management of people with dysphagia following stroke states that they should be offered swallowing therapy at least three times a week, if they are able to participate, for as long as they continue to make functional gains. Therapy could include compensatory strategies, exercises and postural advice [2].	

#### Developing the questions

Using the description of the intervention, we formulate a series of questions based on the innovation description (Table 3). These begin with the most narrowly focused and move to wider category-based questions. These consider innovations in the same category and are key to production of a useful RES where evidence for the innovation is limited. Questions use the PICO(S) approach: defining the Population, Intervention (Innovation), Comparator, key Outcomes and Setting (where relevant) [36]. The eligible study designs will always include existing evidence syntheses or, in their absence, the most relevant primary research design.

**Table 3:** Example: key questions

1. ***Focus on specific innovation:*** What is the evidence for the impact of Phagenyx for key outcomes in people with neurogenic dysphagia compared to other interventions or to usual care? 2. ***Focus on innovation category:*** If there is limited evidence for Phagenyx, what is the evidence for the impact of similar interventions (pharyngeal electrical stimulation) for key outcomes in people with neurogenic dysphagia compared to other interventions or to usual care? 3. ***Focus on wider relevant innovations***: If evidence for pharyngeal electrical stimulation is limited, what is the evidence for the impact of interventions for neurogenic dysphagia more generally?	

If the innovation is an intervention, then the questions will be ones of effectiveness and safety; where the innovation is (for example) a diagnostic test or screening tool, we consider accuracy as well as the impact on participants and health systems of implementing the technology. For complex interventions, each core feature is described and underpins the formulation of several questions. When evaluating evidence for particular components of a complex intervention, we are mindful of the fact that effectiveness in such an intervention may derive not solely from the additive effect of components but from their interaction with each other, as well as with the (often complex) system context [37]. We would therefore consider evidence relating to an individual component to be indirectly relevant to the innovation as a whole. There may be multiple questions of this form (to reflect different populations or comparators, for example). For example, an intervention designed to support inhaler use required separate questions for the populations of people with asthma and people with chronic obstructive pulmonary disease.

Whilst we first focus on effectiveness evidence for the specific innovation being assessed, where this is limited, we will explore evidence for (2) the category of innovations (“innovations like this”) and then (3) wider categories of relevant innovation (e.g. “innovations with a similar aim”). Categories are sometimes not obvious, particularly where the innovation is complex [22–24, 28, 38]. In the case of wider categories, we may ultimately be looking at any intervention with a purpose similar to the index innovation or all interventions for the condition or issue under consideration (see Table 1).

These subsequent questions are designed to ensure that useful evidence can be provided where the evidence for the innovation itself is absent, limited or not directly relevant. Development of these questions involves consultation with key individuals to ensure the focus is relevant. For example, such consultation determined that the widest appropriate question in an appraisal of a particular digital care plan for dementia was “digital care plans for any condition” rather than “care plans for dementia”. These additional questions are designed to be addressed only where the evidence for the first question(s) is considered insufficient. Implementation of the sequential question set is then flexible and sensitive to the nature of the identified evidence. Additional questions are also required when innovations are tests, where the evidence for available treatments should be considered as a whole in the absence of test-and-treat evaluations [31].

#### Types of evidence

Our focus is always on those study designs best able to answer the questions we have developed. We focus on the identification of existing evidence synthesis (systematic reviews) where possible; where this is not possible, we focus on the most informative primary evidence. In the case of most innovations, this is from comparative studies, giving priority to randomised controlled trials. Where appropriate to the questions, we also include diagnostic accuracy or prognostic studies. There are also questions, especially where the focus of an innovation is on patient experience, where mixed methods or qualitative studies will be the most appropriate form of primary evidence.

#### Identifying evidence

We adopt a pragmatic and iterative approach to identifying evidence. This uses an initially narrow focus to maximise relevance and progresses to a broader evidence base as necessary. We search key resources including NICE guidance [39], PubMed and the Cochrane Library, which includes both the Cochrane Database of Systematic Reviews and the Cochrane Central Register of Controlled Trials. We also use reference checking or forward citation searching of relevant evidence syntheses and primary studies.

Where relevant, we increasingly encourage those putting forward an innovation for consideration to provide research evidence, as would be the case with a single technology submission to NICE [40], and we routinely search a commercial sponsor’s website. In some cases, sponsors supply evidence which is unpublished or is published but not peer reviewed. We incorporate this material but are transparent about its status. Our own searches often identify grey literature which is treated in the same way.

Where appropriate, we use subject/domain-specific resources, such as the webpage of a particular Cochrane Group [41] or the ORCHA database of health apps [42]. Where required, we consult with an information specialist. Our decision to consult an information specialist is based on the complexity of the innovation and, where appropriate, the wider categories of interventions.

#### Critical appraisal

We use appropriate methods to critically appraise the different types of evidence we identify. Cochrane reviews are generally considered to represent reliable evidence [43], and we use their summaries and assessments of evidence certainty rather than re-appraising the evidence, unless there are issues around relevance. Where possible with other high-quality systematic reviews, we will also use the existing assessments of evidence from the review. This approach maximises the use of existing high-quality evidence whilst improving timeliness. We consider the quality of non-Cochrane systematic reviews, using the signalling questions from ROBIS as a guide [44]. We consider the possibility of duplication of evidence between multiple evidence syntheses [45].

Where there is no existing evidence synthesis, or we have concerns about the robustness or relevance of a systematic review, we consider primary evidence for the question. We also move to assessing primary research where the existing synthesis has only partially addressed a question, for example because eligibility criteria were narrower. Conversely, where a review has a broader remit, we may look at the included primary studies relevant to our question. In the example of the Phagenyx RES, we looked at the subgroup of RCTs assessing Phagenyx within the Cochrane review of interventions for dysphagia in stroke [46].

Assessment of primary studies considers both the *capacity* of the study design(s) to answer the question and an assessment of the risk of bias in the identified studies of the review to produce *overall* judgements of reliability. Because of our narrow timescale, we do not undertake full assessments but, as with ROBIS, are guided by the domains used. For example, for randomised controlled trials, we are guided by the criteria and considerations of the Cochrane Risk of Bias tool [47]; for other study designs, we consider questions posed by tools such as ROBINS-I, QUIPS, etc. [48, 49].

#### Relationship with GRADE

In forming judgements about the certainty of the evidence, we are guided by the principles of GRADE [21, 50]. GRADE assesses the certainty of evidence through evaluation of several domains in order to produce an assessment of high, moderate, low or very low certainty. The first domain is the risk of bias in the evidence, which we consider as outlined above. This is considered alongside questions of imprecision, inconsistency, direct relevance of the evidence, and publication bias. There are adaptations of GRADE for non-effectiveness questions [51, 52].

Apart from the risk of bias, the domains most relevant to our rapid evidence syntheses are usually imprecision (because of small sample sizes) and indirectness (often a function of context): there is often insufficient evidence to determine inconsistency between studies for the initial questions because there are usually only a small number of studies. The evidence for category-level questions is often of higher certainty than the evidence for the innovation itself; here, the domains of inconsistency (and completeness of evidence (publication bias)) are more likely to be considerations.

We bear in mind that where inconsistency is present (as at the wider category level), this may be a consequence of either—or both—differences in the interventions or the systems in which they are evaluated as well as differences in participants or outcome measures. Whilst some interventions are clearly complex, even apparently simple interventions are frequently implemented into complex health systems and this is especially true of those which would represent changes in patient management [32]. We therefore keep in mind that there may be non-obvious reasons for inconsistent evidence. This especially includes diagnostic and prognostics test, for which we always primarily address a key question about the effect of testing on the people involved and their management [31].

In our RES approach, we explore possible reasons for identified inconsistency and attempt to relate these to the Greater Manchester context. For example, in one RES, we identified a pilot study that reported between-site differences in the impact of an intervention on increasing staff participation in a specific form of mental health assessment. It appeared likely, after considering the qualitative data in the case studies, that this differential was likely to be due to one of the sites being part of a large urban area, whilst the other was very rural [34]. As we noted above, this led to us reporting that the evidence from one site was more directly relevant to our decision problem. Sometimes, as here, the probable cause of inconsistency can become clear; in other cases, we may identify several potential explanations. Where appropriate, we may consult with our RES end-users to establish more details about the implementation context to inform our assessment of the likely importance of identified inconsistency and its relationship to indirectness. Clearly, if inconsistency suggests that some of the evidence is not directly relevant, then this may have implications for the precision of the directly relevant evidence.

Imprecision is usually the consequence of small studies with insufficient participants; this results in wide confidence intervals and effect estimates which would be highly likely to change with further evidence. Indirectness is also often an issue for some or all of the evidence. This may be because of inconsistency (as discussed above). However, because innovations assessed are novel, there is also often only a partial evidence base, where the evidence may be only indirectly relevant to many of the people in question, although directly relevant to the group represented in the studies.

Our considerations of relevance (which GRADE considers as (in)directness) are key to our assessments. In addition to the consideration of indirectness which informs our assessment of the certainty of the evidence, we also consider the relevance of the evidence to the context and health system in which the innovation would be implemented—in this case, Greater Manchester in the UK.

#### Synthesis of the evidence

We use the identified evidence to produce narrative summaries of the evidence for the key questions in the RES. We always summarise evidence for core question(s) relating to the innovation, although we may identify little or no (useful) evidence. We provide a separate answer to each question addressed.

Where possible, we summarise existing evidence syntheses, together with either their existing GRADE assessment or, if these are not available, a judgement based on our assessment of the GRADE considerations. We also provide an assessment of how relevant the evidence from the existing synthesis is to the question.

Where we have been unable to identify relevant existing evidence synthesis, we summarise the primary studies identified. We use a narrative summary to report effect estimates (with confidence intervals) and their certainty and relevance, very rarely would we seek to undertake meta-analysis.

We outline the certainty and relevance of the evidence for each outcome in the question, distinguishing where appropriate the population or subgroup to whom it is directly relevant. So in the Phagenyx population, the evidence is directly relevant to people who have dysphagia following stroke, who represent a subgroup of people with dysphagia. We adopt the GRADE principle of assigning judgements around certainty to a particular outcome rather than at the study level. Where appropriate, we report the evidence for each component of an intervention or intervention bundle (where there is no or very limited evidence for the whole). We provide as nuanced a summary of the evidence as possible, clarifying where evidence has different levels of certainty for different populations, components, or outcomes. An example of a full RES is provided in the Supplementary information.

#### Producing a summary

We provide two levels of summary information, written in non-technical language.

The first provides a single brief summary of the evidence picture and highlights its certainty and relevance (Table 4).

**Table 4:** Example: Headline summary

Phagenyx **may not change clinical outcomes** in people with dysphagia following stroke (low to moderate certainty evidence) but **probably increases the likelihood of decannulation** in people with tracheotomy and dysphagia following stroke (moderate certainty evidence). This is based on randomised controlled trials. Evidence in neurogenic dysphagia in other conditions is limited.	

The second provides a bulleted summary of the certainty and relevance of the evidence for each key question, including (e.g.) nuances of the population to which the evidence is directly relevant (Table 5). This may include aspects of the evidence where relevance to the NHS, or to Greater Manchester, is limited. In both sections, summaries include questions for which we identified no evidence, very limited evidence or very uncertain evidence. The summary follows the approach of the whole evidence synthesis and does not make recommendations to the decision-makers.

**Table 5:** Example: bulleted summary

• Most evidence relates to people with neurogenic dysphagia following stroke. In this population: ∘ There is **low to moderate certainty evidence from RCTs**, including a moderately sized and methodologically strong trial, that Phagenyx **may not change clinical outcomes** in the general population of people with dysphagia following stroke. This is **directly relevant evidence to the UK NHS**. ∘ In people with dysphagia and tracheotomy following stroke, there is **moderate certainty evidence from small but well-conducted RCTs** that **decannulation is probably more likely** in people treated with Phagenyx. This evidence is limited by imprecision but **directly relevant to the UK NHS**. ∘ There is **indirectly relevant evidence** from a **Cochrane systematic review** that, in people with dysphagia following stroke, swallowing therapy of any type probably has no effect on mortality but probably does reduce the length of inpatient stay (moderate certainty evidence) and may reduce the proportion of people with dysphagia (low certainty evidence). Trials of Phagenyx contributed to this much wider review. • There is **limited non-randomised evidence** assessing pharyngeal electrical stimulation in people with dysphagia due to causes other than stroke (people with multiple sclerosis and people in ICU). • Further research may change the findings; the number of people involved is relatively low and new studies could substantially change the results.	

#### Flexibility in structuring the rapid evidence synthesis

As described above, our question series has three possible levels: these relate to (1) evidence for the specific innovation, (2) evidence for innovation category and (3) evidence for wider relevant innovations. Our process involves addressing these questions sequentially, stopping at the point at which we have identified evidence of sufficient certainty and relevance. For the Phagenyx example, suitable innovation-specific (level 1) evidence was identified and no further evidence was required [46, 53]. In another example, a chatbot for mental health [54], there was limited innovation-specific evidence so the search was extended to evidence for the innovation type (level 2) question [55, 56]. For novel innovations that are not part of a wider innovation group, only innovation-specific evidence will be relevant [34].

The use of these question sets allows us to be agile in our approach to RES. Where we consider a multicomponent or bundled innovation, we can rapidly review evidence for the innovation as a whole and, where required, evidence for the innovation components. An example of this is the RES we carried out for RESTORE-2, a tool for care home staff which consists of three key components: identification of “softs signs” of possible physical decline, an early warning score and a structured communication plan. We identified limited evidence for the intervention as a whole [57], so looked at level 1 and 2 questions, as required, for the different innovation components [58–60].

The relevance of evidence reported in the RES is considered during subsequent decision-making, with transparent and cautious extrapolation of indirect data where required. For example, in a RES for an innovation for both people with asthma and people with chronic obstructive pulmonary disease (COPD), we found only randomised evidence for people with asthma [61, 62], and this was extrapolated to people with COPD in the absence of other suitable evidence, but we also considered a level 2 question for people with COPD [63, 64]. This is one of the areas where experience suggests it is most useful for the researcher who produced the RES to be available for consultation during the subsequent decision process, as this allows the relationship between the identified evidence and the local context to be explored in detail.

## Discussion

The need for evidence-informed decision-making is increasingly apparent for a wide range of healthcare organisations in a climate of increasing and competing demands for services. Decision-making is informed by multiple considerations, including costs and opportunity costs, acceptability and existing infrastructure. Whilst it is critical that decisions are informed by evidence, it is also important that this process is both transparent and consistent. As with all rapid evaluation work, there is a necessary trade-off between rigour, speed and available research resources [65].

The RES we undertake are not conducted as an alternative to full systematic reviews, or even more conventional rapid reviews. Rather they represent the introduction into decision-making of some synthesis of existing research evidence. Rapid evidence synthesis itself is widely used in the form of rapid reviews [4], and the use of rapid reviews to inform research, services and policy-making is becoming established [6–10]. However, the development of a clear framework for both the integration of the RES into a rapid local decision-making process and for the production of the RES is, to the best of our knowledge, unique in the field of innovation adoption.

The framework presented here is grounded in the GRADE evidence to decision approach as well as previous work in evidence briefing services [16, 20]. It utilises the principles of this approach to support researchers who need to rapidly identify, assess and synthesise evidence from existing evidence syntheses and other sources in order to support immediate, real-world, healthcare decision-making processes. These processes are multidimensional, taking account of evidence alongside stakeholder views, system constraints and financial considerations. We have found that developing and using this framework provides improved transparency about the evidence base for innovations, including the limitations and gaps, to inform pragmatic decisions about implementation and future evaluation needs. There is also transparency and consistency about the process used to generate the evidence synthesis and the limitations on this which are necessary for the rapidity obtained.

The framework is intended for use where there may be relatively limited evidence available for the innovation, as well as where more research is available. It is envisaged that this approach is used by researchers from organisations involved in decisions about innovation adoption, so that queries are rapidly resolved in the innovation description and question formulation stages. In our process, researchers attend relevant meetings, enabling them to answer queries and discuss issues with stakeholders and decision-makers which arise from the RES. The RES does not therefore exist only as a stand-alone document but as part of a broader integration of relevant research evidence in the decision-making process. This may distinguish the process we present from other evidence briefing services which have tended to be externally commissioned [6, 16].

### Strengths and limitations of the process

The principal limitation of the process is the necessary trade-off between rapidity and both comprehensiveness and rigour which all rapid evaluation confronts [65]. This is the case both in evidence synthesis and in primary research [66, 67]. These trade-offs are sometimes explicit—as is the case with the NICE Digital Health Framework, which is particularly relevant to the significant proportion of our evaluations which relate to digital health innovations [68]. In this instance, we use a transparent and structured process to make the trade-offs for rapidity of evidence synthesis explicit. However, it is known that rapid review processes in general may produce different results to full systematic reviews. This is the case where processes are more complex than those employed in this rapid evidence synthesis process [66, 69, 70].

We have not formally compared the results of our RES with those of a systematic review: although the need for a full systematic review may be identified, there is often insufficient time or resource to commission a further piece of evidence synthesis for the current decision problem, and this limitation is flagged. Where a systematic review is identified as being a high priority, the scope for this can be explored within the wider ARC-GM. We are currently developing a full systematic review informed by the RES process, although with a narrower review question; we plan to compare the full review findings with those of the RES once it has been completed.

It is likely that we will not identify all relevant evidence for some questions, particularly broader category-level questions. This is particularly likely to be the case where we do not identify any relevant evidence syntheses and are summarising primary studies. The iterative process we use, which includes citation searching and a saturation-based approach, mitigates this risk, as does the focus on existing evidence syntheses. The fact that the RES is produced by a single researcher also makes it necessarily vulnerable to bias and error. The potential for error may be mitigated by the researcher being relatively experienced in evidence synthesis [71]; a possible adaption to the process would be to incorporate checks by a second researcher, with a concomitant cost in time and resources.

We are especially conscious of the difference that the involvement of an information specialist or medical librarian may make to a systematic review [72, 73], and we have been able to informally assess the impact of their input on the RES. In three instances, we conducted an initial search but, due to time constraints, were only able to consult the information specialist after we had assessed our search results. Across these assessments, their amendments to our searches resulted in the inclusion of one additional study in one RES, which did not make a substantive difference to the findings; in the other two, no changes were made as a result of their involvement. We think it is likely that the impact of involving an information specialist may be less critical because in many cases we are searching for existing systematic reviews, and these may be better indexed than other types of publications [74].

We also acknowledge that searching a limited selection of databases (with or without specialist input) can have an impact on the findings of an evidence synthesis [75]. We accept that we may miss some relevant evidence: because the RES is not a systematic review, it is unlikely to be exhaustive. However, we never search only a single database, and our database searches are supplemented with other methods such as citation searching. Iterative processes as we look at wider questions mean we adopt elements of a saturation approach, which may also mitigate the risk of missing key pieces of evidence; again the fact that we are searching for evidence syntheses for some stages of the RES may also mitigate this through better indexing [74]. Where we are searching for primary evidence, the fact that we are considering specific innovations rather than broader interventions may also mean that the combination of limited database searches and the use of a single reviewer makes less difference to the outcome [76].

We have considered the potential for information supplied by commercial sponsors to have undue influence on the process. As is the case in our experience of the NICE single technology appraisal approach [40], only very rarely have commercial sponsors submitted published material eligible for inclusion which has not been identified by our own searches. Where newly published material is brought forward, we use reference checking and identification of key terms to augment our database searches. Material which is unpublished but relevant is handled in the way in which personal communications from authors would be within a systematic review and is clearly identified as such. Such material is usually interim or additional analyses from a study we have previously identified and is considered as additional material for that study. Where sponsors supply evidence which is not relevant to the decision problem, we very briefly summarise this, noting the reasons we have excluded it from our evidence synthesis. 

The approach to rapid evidence synthesis outlined here has the advantage that it can be undertaken very rapidly: it is designed to be undertaken in the 2 weeks between the initial decision that an innovation has potential merit and a subsequent meeting, where an adoption decision will be made. This represents a very short timescale, even for evidence briefing production [7–9, 19], and a much shorter timeframe even than most rapid reviews [3]. In maximising the use of existing evidence synthesis wherever possible, it is an efficient process which minimises research waste.

The RES approach outlined is designed to be extremely flexible, both in terms of the questions which can be addressed and the process of answering those questions; it is an iterative and pragmatic process whereby researcher judgement can be used to refine the approach at every stage. This means it is easily adapted for the assessment of a wide range of innovations: those we have so far assessed include medical devices, screening and prognosis testing, bundled service process interventions and digital mHealth apps.

### Implementation and evaluation

Our approach to rapid evidence synthesis has been developed and implemented in a real-time context of decision-making around the adoption of innovative health technologies. Key stakeholders in this decision-making process have found that it is sufficiently timely and flexible to be a useful input and have engaged actively with its production and interpretation. There is substantial interest from other ARCs and AHSNs in implementing a similar process; creating a common resource database of RES undertaken by any organisation would further minimise research waste and improve evidence-informed decision-making. We are exploring options to enable this, including making some RES publicly available. Although we consider local relevance, the RES first consider the relevance of evidence to the NHS in England, meaning that they are also relevant to other regional decision-makers.

We track the progress of innovations for which we have undertaken RES; decisions to date have included adoption, requests for further information from the sponsor, and decisions not to progress. For innovations now adopted for roll-out, the RES can inform subsequent evaluation questions. Published evaluations of the use of evidence briefings are limited [19], and we are considering possible approaches to evaluating our use of RES.

## Supplementary Information

**Additional file 1.** The NIHR ARC-GM and Health Innovation Manchester approach to rapid evidence synthesis to support health system decision making.

**Additional file 2.** Rapid evidence synthesis: Phagenyx.

## Data Availability

The framework for this methodology is publicly available on OSF: https://osf.io/hsxk5/. It is also cited in the bibliography and is available as supplementary information to this paper. Full copies of all rapid evidence syntheses produced are available on reasonable request to the corresponding author; an example is included in the supplementary information.
