# Supplementary material for: Rapid evidence synthesis to enable innovation and adoption in health and social care
Source: Syst Rev. 2022 Nov 23;11:250. doi: 10.1186/s13643-022-02106-z (PMC9682764; doi:10.1186/s13643-022-02106-z)
Supplement: Supplementary file 1 — Additional file 1. The NIHR ARC-GM and Health Innovation Manchester approach to rapid evidence synthesis to support health system decision making. [file 13643_2022_2106_MOESM1_ESM.docx]

The NIHR ARC-GM and Health Innovation Manchester approach to rapid evidence synthesis to support health system decision making

Decisions about how to proceed with new health innovations should be informed by knowledge of the current research about the innovation: *How much research is there?* *Should we believe the results?* *Can we apply the results here?*

We undertake an initial rapid appraisal to answer these questions. The nature of the evidence will be slightly different for every innovation: broad principles and approaches are described here as a guide.

By innovation we mean any technology, device, procedure, set of behaviours, routine, or way of working that is new to the Greater Manchester context.

**Our approach – and what makes it rapid?**

We produce an initial rapid assessment of available evidence in time for incorporation in next weekly or fortnightly meeting to decide whether to progress the innovation. The process requires that we:

- **Describe the innovation:** Describe the nature and purpose of the innovation. Is the innovation an intervention or something else such as a screening tool or diagnostic or other test? Are there are any existing alternatives and, if so, what are they?
- **Formulate questions** based on key characteristics and aims of innovation: consider the PICO (Population, Intervention, Comparator, Outcomes). Use sequential questions to focus on available evidence in order of relevance and how well-developed it is. Do not seek stakeholder consultation at this stage.
- Use a **highly focused approach to searching;** use iterative methods to widen the search to answer broader questions and preferentially identify the most reliable available evidence (starting with existing evidence synthesis). Consult an information specialist if possible. Search key sources: use the Cochrane Library and Medline plus other sources such as the NICE website. Stringently screen results for relevance, focusing first on identifying any existing evidence syntheses.
- Begin with an initially narrow focus in order to provide maximise relevance. Progress to a broader evidence-base **as necessary;** accepting trade-offs between speed, comprehensiveness and relevance.
- **Assess the evidence:** For each question appraise **the most reliable types of evidence available**. **For intervention-related questions**, start with systematic reviews, progressing to RCTs (where these are feasible for the question) in the absence of reviews or to update the evidence. If needed, move to consider other comparative designs before considering uncontrolled studies**.**
- **Evaluate the evidence** using appropriate methods such as the Cochrane tool for assessing risk of bias in RCTs and GRADE for certainty of a body of evidence. For non-RCT evidence there are other appraisal tools and adaptations of GRADE. **Consider its relevance** to the Greater Manchester context in terms of service model and populations.
- **Summarise the evidence:** form a judgement on the confidence which can be placed in it (being explicit about uncertainty) and its relevance for the key questions
- Complete the process in a **narrow time-window** (we aim for 0.5 to 1 day) to support decision-making. **Prepare the groundwork** for further evaluation (e.g., questions that should be addressed in proof-of-value or deployment at scale).

# Getting the question right

Typically, decision-makers want to know: *Does this innovation work? Is it safe? Are the benefits worth the costs?* We have developed a rapid approach to summarising the existing research evidence. These rapid evidence syntheses then inform decisions about innovation adoption in the GM innovation pipeline.

We identify the most important questions to be asked of the evidence. Many innovations are best considered as interventions or treatments. Therefore, the key questions will ask about the impacts of the innovation on outcomes (impacts may be beneficial or harmful). Questions are typically focused using the PICO approach: **Population**; **Intervention**, **Comparator**, **Outcomes**.(1) The question would then be: “*What are the effects in the* ***population of interest*** *on* ***important outcomes*** *of the* ***specific intervention*** *compared with* ***other interventions or usual care?*”** [Box 1, Q1]

There may be multiple questions (to reflect different populations or comparators, for example).

We also adopt a sequential approach to evidence finding so, although we focus in the first instance on the specific innovation, a wider scope may sometimes be required. For example where there isn’t much research about a specific intervention, there may be evidence about related interventions (the same ‘class’ of interventions, like different version of apps with shared features) so we may ask: “*What are the effects* ***on important outcomes*** *of* ***interventions like this*** *in* ***people like this*** *compared with* ***other interventions or usual care****?*” [Box 1, Q2-Q4]

**BOX 1 Example of Key Questions 1**

***Hypothetical innovation: a new mobile phone app “InnovationApp” that aims to help older people living in the community exercise safely.***

Q1: What is the effect of using **InnovationApp** on outcomes such as hospital admissions, fitness and quality of life in older people living in the community?

***Potential additional questions (dependent on evidence found for the previous question)***

Q2: What is the effect of **any app for supporting exercise** on outcomes such as hospital admissions, fitness and quality of life in older people living in the community?

Q3: What is the effect of **any digital intervention which aims to support exercise,** on outcomes in older people.

Q4: What is the effect of **any intervention to support exercise** on outcomes in older people.

Additional questions will be needed for specific types of innovations. For example, in the case of diagnostic tests we would consider: “*How* ***accurate*** *is the test/method in predicting or diagnosing the* ***condition of interest****? What is the additional (marginal)* ***value of information*** *from using the test?*” [Box 2, Q1] Where appropriate we would also consider the accuracy of tests/methods in the same class as the intervention. [Box 2, Q2] Importantly these questions would always be considered in the context of an effectiveness and safety question: what is the **impact** of using this test/method on **outcomes for people**? [Box 2, Q3] There is an additional question “How **effective** are **interventions for preventing or treating the condition** the test aims to predict or detect?” [Box 2 Q4] This is important because testing should always be considered in terms of the consequences (or value) of particular test results for both individuals and health services.

**BOX 2 Example of Key Questions 2**

***Hypothetical Innovation: A new test “ControlD2” to improve the identification of people with poorly controlled type 2 diabetes***

Q1 In **people with diagnosed type 2 diabetes** how ***accurate*** is C**ontrolD2** at identifying people whose diabetes is not well controlled, **compared with current best practice**?

Q2 In **people with diagnosed type 2 diabetes** how ***accurate*** are **methods like this** at identifying people whose diabetes is not well controlled, **compared with current best practice**?

Q3 What is the **impact** of using **ControlD2** (**or methods like this**) for identifying people with diabetes which is not well controlled on **outcomes** such as blood glucose, incidence of complications of diabetes and requirement for unscheduled medical care, **compared to current best practice**?

Q4 What is the **impact on important outcomes** of **interventions** for people with type 2 diabetes whose diabetes is not well controlled?

**of key questions**

*Hypothetical Innovation: A test (method) to improve the identification of people with poorly*

# Searching for the research

Once the questions are clearly posed, we search for the research evidence that can answer the question(s). We look for published research using rapid searches of key databases and websites, focusing first on finding any existing syntheses of the research. We also look at evidence submitted by the innovation sponsor (often a manufacturer); or reported on the sponsor’s website/in other available documentation. Typically we search the Cochrane Library (2) which includes the Cochrane Database of Systematic Reviews (CDSR) and Ovid Medline which includes PubMed.(3) Key websites are the UK National Institute for Health and Care Excellence (NICE) (4) and the sponsor’s website. Other databases and sources can be tailored to the intervention and we may also use reference checking of identified publications.

# Types of research

Because we are producing a rapid assessment we look first for **existing reliable summaries** of the relevant research, known as **systematic reviews**, which are most helpful to decision makers. We also look for relevant guidance from bodies such as the UK National Institute for Health and Care Excellence (NICE).(4)

The research design most likely to give the best answer about the effects of an intervention is the **randomised controlled trial (RCT)** because, when well conducted, it is the fairest comparative test. Systematic reviews of evidence for interventions will often include only RCTs but may include other study types.

If there are no existing systematic reviews which answer our question(s) we will look for RCTs.

For innovations which are not interventions we look for specific types of studies which are designed to assess these. These include studies of diagnostic test accuracy [Box 2, Q1, Q2] but we would always also look for studies assessing the impact of using tests or screening tools on outcomes for patients and these would ideally be RCTs [Box 2, Q3].

For all innovations, if there are no relevant systematic reviews or RCTs then we consider other forms of comparative evidence. Such evidence cannot provide the same level of certainty as high quality, randomised evidence which is the best way to answer many questions where the impact of an intervention on outcomes for people is concerned.

However for some types of innovation, such as methods of service delivery or organisation, or introduction of new policies it is rare for RCTs to be feasible, or indeed possible. Existing systematic reviews of these types of innovations will include other types of evidence such as reports of natural experiments or comparative longitudinal data; where there are no relevant existing syntheses we look for this type of primary research report.

Studies without a contemporaneous comparator, or without any comparator, are only considered in the absence of comparative evidence. Other types of research (for example laboratory or pre-clinical studies) may be useful in some circumstances, in the absence of clinical studies or in addition to them. For some interventions qualitative evidence may be valuable in helping to evaluate impact.

# Assessing the reliability of the research results

We then have to assess whether the existing research was conducted in such a way that we can have confidence in its results.

We use a recognised approach following the guidance of the Cochrane Collaboration. If we identify a Cochrane review or another systematic review which reports using the same rigorous approach then we can usually have confidence in its results. Many systematic reviews, and all recent Cochrane reviews, use the GRADE method to rate the evidence from high certainty to low certainty and we will use these judgements where available.(5)

Where we do not have systematic reviews but have other types of evidence we use a rapid version of the GRADE approach to help us form this type of judgement about the reliability of the evidence. If we are assessing RCTs which have not been included in an existing systematic review we use the Cochrane risk of bias tool to look at how the methods used could affect the risk of biased results from each study;(6) other tools are available for different types of study.(7) We also look at whether the different studies have similar findings (consistency of the evidence), how clear the reported effects were and how relevant the findings are to our question. We do not undertake meta-analysis in our rapid syntheses but provide a narrative interpretation and summary of the results of different studies. There are adaptations of GRADE available for looking at other types of evidence.(8) There is also detailed guidance and resources for assessment of studies of test accuracy.(9)

# Assessing the relevance of the research results

We consider the relevance of the research in two (overlapping) ways. Firstly we look at whether there is evidence which assesses the innovation itself and whether the research assesses it in the way in which it would be used if it were implemented: does it look at the same type of people and the same sorts of ways of assessing impacts, is it being compared to something which might be used in practice?(5)

Secondly we consider the relevance of the research to the Greater Manchester. In practice this is likely to be relevance to NHS context but it may also consider more locally relevant factors such as whether an assessment was conducted in a highly urbanised or a rural area and whether the population of the study reflects that in Greater Manchester as well as the UK more broadly. Particular organisational features of the Greater Manchester health and social care context may be relevant (e.g. DevoManc and presence of highly integrated teaching hospital NHS trusts). Generally we only undertake an appraisal where an innovation is not part of a national priority stream.

# Forming a judgement

We consider the type, quality, and relevance of the evidence in order to form an overall judgement regarding what it is telling us (the *amount* of evidence is part of the assessment of quality). Both the quality of the research evidence and its relevance can range from high to low. If there are **multiple questions** then the quality and relevance may be different for each question. For example, evidence may be high quality but only directly relevant to a specific group of people, or there may be very limited evidence of low relevance for the specific intervention but moderate quality, highly relevant evidence for the class of interventions.

Example answers to the key questions as a result of the assessments of reliability and relevance are shown. These are integrated into an overall summary assessment. [Box 3]

**BOX 3 Example of summary answers to Key Questions 1**

*Hypothetical innovation: a new mobile phone app “InnovationAPP” that aims to help older people living in the community exercise safely.*

Q1: There is no evidence on the impact of using InnovationAPP on outcomes such as hospital admissions, fitness and quality of life in older people living in the community.

Q2: There is some limited evidence from controlled before-and-after studies that some apps for supporting exercise may improve fitness and quality of life compared to usual care, but there is no evidence for their impact on hospital admissions for older people living in the community.

Q3: There is moderate certainty evidence from a systematic review that some digital interventions which aim to support exercise, probably improve specific measures of fitness in older people living in the community but probably have little or no effect on hospital admissions.

Q4: There is high certainty evidence from several systematic reviews that some interventions to support exercise reduce hospital admissions in older people in residential care but the evidence is less clear for those living in the community. Positive effects on other outcomes are seen in both groups.

**Example Summary of Evidence**

There is no evidence for the impact of InnovationApp on outcomes in older people living in the community and only limited evidence for other apps designed to support exercise in this group. Relevant evidence shows that digital interventions probably improve fitness and quality of life but not outcomes related to healthcare use. There is good evidence that exercise support interventions, considered generally, improve outcomes, but improvements in healthcare use are clearly seen only in older people in residential settings so may not be relevant to this population.

# Time and resources

- Close working and co-location of the NIHR Applied Research Collaboration for Greater Manchester (NIHR ARC GM) and Health Innovation Manchester facilitates clear understanding of the requirements for each innovation assessment.
- Average of one day of researcher time for each rapid evidence synthesis. This varies depending on the volume and nature of the available evidence. Using a researcher with significant experience in critical appraisal and evidence synthesis reduces time taken.
- Search time is limited as searches are brief and iterative, this averages 1-2 hours but may vary depending on the innovation. Using an information specialist helps to reduce time taken. Access to reported research (published and unpublished) from the sponsor reduces time spent identifying research from other sources.
- Access to publicly available websites, databases and open access research; access to research reported in subscription journals through the University of Manchester.

**30 July 2020**

**References**

1. National Institute for Health and Care Excellence (NICE). The guidelines manual.

<https://www.nice.org.uk/process/pmg6/chapter/developing-review-questions-and-planning-the-systematic-review>. Accessed April 2020

1. Cochrane Library. <https://www.cochranelibrary.com/> Accessed April 2020
2. Medline (PubMed) <https://pubmed.ncbi.nlm.nih.gov/advanced/>) Accessed April 2020
3. National Institute for Health and Care Excellence (NICE) website <https://www.nice.org.uk/>. Accessed April 2020
4. Guyatt G, Oxman AD, Akl EA et al. GRADE Guidelines: 1. Introduction-GRADE Evidence Profiles and Summary of Findings Tables. Journal of Clinical Epidemiology 2011; 64 (4): 383-94. <https://pubmed.ncbi.nlm.nih.gov/21195583/>
5. Higgins JPT, Savović J, Paget MJ et al. Cochrane Handbook for Systematic Reviews of Interventions Version 6, 2019. Chapter 8: Assessing risk of bias in a randomized trial. <https://training.cochrane.org/handbook/current/chapter-08>. In: Higgins JPT, Thomas J, Chandler J, Cumpston M, Li T, Page MJ, Welch VA (editors). Cochrane Handbook for Systematic Reviews of Interventions version 6.0 (updated July 2019). Cochrane, 2019.
6. Reeves BC, Deeks JJ, Higgins JPT, Shea B, Tugwell P, Wells GA. Chapter 24: Including non-randomized studies on intervention effects. In: Higgins JPT, Thomas J, Chandler J, Cumpston M, Li T, Page MJ, Welch VA (editors). Cochrane Handbook for Systematic Reviews of Interventions version 6.0 (updated July 2019). Cochrane, 2019. <https://training.cochrane.org/handbook/current/chapter-24>
7. Schünemann HJ, Cuello C, Akl EA, et al. GRADE guidelines: 18. How ROBINS-I and other tools to assess risk of bias in nonrandomized studies should be used to rate the certainty of a body of evidence. J Clin Epidemiol. 2019;111:105‐114. doi:10.1016/j.jclinepi.2018.01.012
8. Cochrane Screening and Diagnostic Tests Methods Group. <https://methods.cochrane.org/sdt/welcome>. Accessed June 2020.
