# Supplementary material for: Rapid evidence synthesis to enable innovation and adoption in health and social care
Source: Syst Rev. 2022 Nov 23;11:250. doi: 10.1186/s13643-022-02106-z (PMC9682764; doi:10.1186/s13643-022-02106-z)
Supplement: Supplementary file 2 — Additional file 2. Rapid evidence synthesis: Phagenyx. [file 13643_2022_2106_MOESM2_ESM.docx]

**Rapid evidence synthesis: Phagenyx**

**Summary**

Phagenyx **may not change clinical outcomes** in people with dysphagia following stroke (low to moderate certainty evidence) but **probably increases the likelihood of decannulation** in people with tracheotomy and dysphagia following stroke (moderate quality evidence). This is based on randomised controlled trials. Evidence in neurogenic dysphagia in other conditions is limited.

- Most evidence relates to people with neurogenic dysphagia following stroke. In this population:
  - There is **low to** **moderate certainty evidence** **from RCTs**, including a moderately sized and methodologically strong trial, that Phagenyx **may not change clinical outcomes** in the general population of people with dysphagia following stroke. This is **directly relevant evidence to the UK NHS**.
  - In people with dysphagia and tracheotomy following stroke there is **moderate certainty evidence** **from small but well-conducted RCTs** that **decannulation is probably more likely** in people treated with Phagenyx. This evidence is limited by imprecision but **directly relevant to the UK NHS**.
  - There is **indirectly relevant evidence** from a **Cochrane systematic review** that, in people with dysphagia following stroke, swallowing therapy of any type probably has no effect on mortality but probably does reduce length of inpatient stay (moderate certainty evidence) and may reduce the proportion of people with dysphagia (low certainty evidence). Trials of Phagenyx contributed to this much wider review.
- There is **limited non-randomised evidence** assessing pharyngeal electrical stimulation in people with dysphagia due to causes other than stroke (people with multiple sclerosis and people in ICU).
- Further research may change the findings; the number of people involved is relatively low and new studies could substantially change the results.

**Search**

We searched PubMed and the Cochrane Library which includes the Cochrane Database of Systematic Reviews and the Cochrane Central register of Controlled Trials (CENTRAL). We also evaluated information supplied by the sponsor (Phagenesis) and searched their website as well as the website of NICE. Search terms included “Phagenyx”, “Pharyngeal electrical stimulation” and “neurogenic dysphagia”. Searches were conducted on 22 and 23 July 2020

**Description of the intervention**

Phagenyx is a device which is designed to reduce neurogenic dysphagia (dysfunction of eating).[1] This is dysphagia arising from the disruption of any of the neurological systems or processes involved in the execution of a coordinated safe swallow and occurs in people following stroke and in other conditions such as multiple sclerosis which impact muscle control. Dysphagia also occurs in people who have undergone sustained intubation for any reason. Phagenyx is classed as a pharyngeal electrical stimulation intervention and comprises a base station and a treatment catheter. It is applied over a period of days.

NICE guidance on the management of people with dysphagia following stroke states that they should be offered swallowing therapy at least three times a week, if they are able to participate, for as long as they continue to make functional gains. Therapy could include compensatory strategies, exercises and postural advice.[2]

**Key questions**

1. What is the evidence for the impact of Phagenyx for key outcomes in people with neurogenic dysphagia compared to other interventions or to usual care?
2. If there is limited evidence for Phagenyx, what is the evidence for the impact of similar interventions (pharyngeal electrical stimulation) on for key outcomes in people with neurogenic dysphagia compared to other interventions or to usual care?
3. If evidence for pharyngeal electrical stimulation is limited, what is the evidence for the impact of interventions for neurogenic dysphagia more generally?

**Phagenyx**

There are several randomised controlled trials (RCTs) of the use of Phagenyx in people who have been diagnosed with neurogenic dysphagia following stroke**.** There are also RCTs in people who have a tracheotomy following stroke and a small trial in people who have dysphagia due to multiple sclerosis.

**People with dysphagia following stroke**

Four trials with a total of 214 participants comparing Phagenyx to no or sham treatment were included in a wider Cochrane systematic review of treatments for swallowing difficulties in people with recent stroke.[3] An earlier individual participant data (IPD) meta-analysis included three of these trials (73 participants).[4]

The Cochrane review found that swallowing therapy in general, compared to no treatment or control conditions probably had no effect on mortality but probably did reduce length of inpatient stay (moderate quality evidence) and may reduce the proportion of people with dysphagia (low quality evidence). Other outcomes showed no differences or were represented by very low quality evidence (this included penetration aspiration and chest infections/pneumonia).[3]

RCTs evaluating Phagenyx contributed to the following outcomes: case fatality at end of trial; length of stay; dysphagia at end of trial; swallowing ability; penetration aspiration score; chest infection or pneumonia; pharyngeal transit time; institutionalisation; nutritional status (albumin). The contribution of the Phagenyx studies to these analyses varied, and in some case represented very small numbers of participants. This means that the extent to which the results are directly relevant to the effects of Phagenyx varies. Data on other outcomes is only indirectly relevant (in the sense that it relates only to other types of swallowing therapy interventions). In each outcome the subgroup of studies assessing Phagenyx showed an effect estimate where the 95% confidence intervals included the possibility of both benefit and harm as well as no effect. The Cochrane review did not identify subgroup differences for most outcomes, based on the eight different types of intervention considered. However, we briefly describe the evidence from the included RCTs of pharyngeal electrical stimulation. [5-7]

The largest trial (STEPS) is a phase III comparison of Phagenyx with a sham treatment. [5] This recruited 162 people with clinical dysphagia following recent stroke across five European countries; the largest number of participants were from the UK (62%). The primary outcome was penetration aspiration. Other outcomes were clinical measures of swallowing, feeding status, dependency, pneumonia and adverse events. Participants were followed up for 12 weeks. The trial did not find a difference in either the primary or secondary outcomes. The authors stated that participants may have been undertreated.

The trial used strong research methodology in assigning participants to active or sham treatments. Participants and outcome assessors but not trial personnel were blinded to treatment allocation. A statistical analysis plan was published and there was an independent data monitoring committee; an amendment to primary outcome was made before data were unblinded. Withdrawals were clearly documented, the greatest number of people for whom there were no data were participants who did not receive treatment; this may be relevant to participant tolerance for treatment or to other factors. Evidence from this trial is likely to be both relevant and at low risk of bias; overall this is moderate certainty evidence that there may be little or no difference between the treatment groups for the outcomes assessed. Three other trials, one of which was dose finding, recruited a much smaller number of people.[6,7]*

We identified an ongoing RCT which is still recruiting (planned recruitment 225 participants who have dysphagia following stroke).[8] The Cochrane review also identified a very large number of ongoing studies, some of which may be relevant to this rapid evidence synthesis.

*Two trials are reported in a single paper.

**People with dysphagia and tracheotomy following stroke**

A subsequent RCT [9] enrolled people with neurogenic dysphagia who had undergone tracheotomy following stroke. This randomised 69 participants to Phagenyx or sham treatment and assessed a primary outcome of readiness for decannulation (detailed criteria for this were reported). Participants were in Germany, Austria and Italy. The trial was stopped early by an independent data monitoring board for efficacy based on the primary outcome. Early stopping in a small trial can be problematic; use of independent data monitoring mitigates the risk of artefactual results. Participants were followed up for 90 days. Methodologically the trial appeared well-conducted although the nature of the primary outcome resulted in a complex set of secondary outcomes and participant flow. A number of deaths were reported but were considered unrelated to the intervention; adverse events did not differ between the groups. As the trial authors note the principal limitation of this study is its small size which makes it more likely that findings will change with subsequent research.

Another smaller RCT in this population of people with post-stroke tracheotomy was not included in the Cochrane review because of lack of relevant data at the time of assessment.[10] [Suntrup] This randomised 30 participants in a 2:1 ratio to Phagenyx or sham treatment. The primary outcome was decannulation. Participants in the intervention group were more likely to be able to undergo decannulation that those in the control group; secondary outcomes did not show differences between groups; these included measures of length of stay. The study was undertaken in Germany and reported reasonably good methodology.

These trials represent moderate certainty evidence and appear directly relevant to the NHS context.

**People with neurogenic dysphagia due to conditions other than stroke**

A small RCT enrolled 20 people with dysphagia due to multiple sclerosis; they were randomised to real or sham stimulation using pharyngeal electrical stimulation. The device used was not reported as being Phagenyx. [11] The primary outcome was variation in the Penetration Aspiration Scale; pre-post benefits were reported for the real group but not the sham group; between-group differences were not reported but the data appear likely to support some difference.

A pilot study looked at use of pharyngeal electrical stimulation (not reported whether Phagenyx was used) in people in ICU. [12] This did not appear to be an RCT but used a matched control group. The intervention was delivered while participants were intubated and outcomes were pneumonia and need for reintubation; both of these were reported to be statistically significantly less frequent in the people in the intervention group. Because of the study design and the limited reporting of methods it is difficult to determine whether differences in outcomes were due to the intervention. Another study in a small group of critical care patients did not have any control group.[13]

We also identified a trial registry record for a European registry for the use of Phagenyx in participants with Parkinson’s disease or multiple sclerosis as well as stroke; the results are noted as overdue.[14]

**Gill Norman 28 July 2020**

**References**

1. Phagenesis.com <https://www.phagenesis.com/products>. Accessed 22 July 2020
2. National Institute for Health and Care Excellence (NICE). Stroke rehabilitation in adults. Clinical guideline CG162. 2013. <https://www.nice.org.uk/guidance/cg162/chapter/1-recommendations>. Accessed 23 June 2020.
3. Bath PM, Lee H, Everton LF. Swallowing therapy for dysphagia in acute and subacute stroke. Cochrane Database of Systematic Reviews 2018, Issue 10. Art. No.: CD000323. DOI: 10.1002/14651858.CD000323.pub3
4. Scutt P, Lee HS, Hamdy S, Bath PM. Pharyngeal Electrical Stimulation for Treatment of Poststroke Dysphagia: Individual Patient Data Meta-Analysis of Randomised Controlled Trials. *Stroke Res Treat*. 2015; 2015:429053.
5. Bath PM, Scutt P, Love, J, Clave P, Cohen D, Dziewas R. Pharyngeal Electrical Stimulation for Treatment of Dysphagia in Subacute Stroke. Stroke 216; 47 (6) 1562-70.
6. Jayasekeran V, Singh S, Tyrrell P, et al. Adjunctive functional pharyngeal electrical stimulation reverses swallowing disability after brain lesions. *Gastroenterology*. 2010;138(5):1737-1746.
7. Vasant DH, Michou E, O'Leary N, et al. Pharyngeal Electrical Stimulation in Dysphagia Poststroke: A Prospective, Randomized Single-Blinded Interventional Study. *Neurorehabil Neural Repair*. 2016;30(9):866-875. doi:10.1177/1545968316639129
8. Pharyngeal Electrical Stimulation Evaluation for Dysphagia After Stroke (PhEED). ClinicalTrials.gov Identifier: NCT03358810 <https://clinicaltrials.gov/ct2/show/NCT03358810>. Accessed 22 July 2020
9. Dziewas R, Stellato R, van der Tweel I, et al. Pharyngeal electrical stimulation for early decannulation in tracheotomised patients with neurogenic dysphagia after stroke (PHAST-TRAC): a prospective, single-blinded, randomised trial. *Lancet Neurol*. 2018;17(10):849-859.
10. Suntrup-Krueger S; Marian T; Schröder JB. et al. Electrical pharyngeal stimulation for dysphagia treatment in tracheotomized stroke patients: a randomized controlled trial. Intensive Care Medicine 2015; 41 (9): 1629-37
11. Restivo DA, Casabona A, Centonze D et al. Pharyngeal Electrical Stimulation for Dysphagia Associated with Multiple Sclerosis: A Pilot Study a Brain Stimulation 2013; 6 (3) 418-23
12. Koestenberger, M., Neuwersch, S., Hoefner, E. et al. A Pilot Study of Pharyngeal Electrical Stimulation for Orally Intubated ICU Patients with Dysphagia. Neurocrit Care 32, 532–538 (2020).
13. Wallace S, Sloan L. Efficacy of Pharyngeal Electrical Stimulation for treatment of dysphagia in critical care patients. NMAHP Conference 2019.
14. A European registry to observe the use of the Phagenyx devices for the treatment of patients with swallowing difficulties. <http://www.isrctn.com/ISRCTN87110165>. Accessed 23 July 2020
